# P02-007 - Childhood autoinflammatory disorders in Qatar

**DOI:** 10.1186/1546-0096-11-S1-A114

**Published:** 2013-11-08

**Authors:** B Aladbe, A Aly, R Taha, H Elshanti, T Moussa, F Al Amry, B Fathalla

**Affiliations:** 1 Pediatrics/Rheumatology, Hamad General Hospital, Doha, Qatar; 2 Pediatrics, Hamad General Hospital, Doha, Qatar; 3 Medical Genetics, Shafallah Medical Genetics Center, Doha, Qatar

## Introduction

A multi-ethnic background with high rates of consanguinity characterizes the population living in Qatar. Understanding the uniqueness of clinical presentation of autoinflammatory disorders (AID) in this population will enhance our knowledge in regards to the spectrum of clinical phenotype and prevalence of mutations in our region.

## Objectives

To report the clinical and genetic profile of children with AID from the only childhood rheumatology center in Qatar over the past 5 years.

## Methods

A retrospective review of medical records.

## Results

Familial Mediterranean Fever: 30 symptomatic children, 9 asymptomatic carriers, and 21 adult relatives were included. Among symptomatic children, the male to female ratio was 1:1, 19 were Arabic, 8 were Persian, and 3 were Turkish/Arabic. Median age at first symptoms was 5 years (range 1 – 16 years). Most common manifestations included recurrent abdominal pain and fever (n=25), arthralgia (15), chest pain (4), arthritis (3), oral aphthouses (3), erysipelas (1), and recurrent pyogenic arthritis (1). Other features include anemia (4), hypothyroidism or hyperthyroidism (2), and renal failure due to membranoproliferative glomerulonephritis (1). Response to colchicine was good (23) or partial (2); 4 others are not yet started and 1 was lost follow-up. A 23 member four-generation family of Persian ethnicity was followed showing variable severity of clinical manifestations, severe pustulosis and psoriasis. Out of the expected 34 *MEFV* mutant alleles (17 probands), only 25 were identified while 9 were unidentified. Of the 25 MEFV mutations M694V (12), E148Q (5), E167D/F479L (2), V726A (2), M694I (2), N599D (1), and M680I (1).

Hyper-IgD syndrome group includes one Arabic family: parents and 3 siblings are carriers of V377I/- *MVK* mutation. Two symptomatic siblings are homozygote for V377I *MVK* mutation. All members have complex *MEFV* mutations. Detailed clinical and genotype characteristics are reported separately due to exceptionality of such combination.

One boy with Pyogenic Arthritis, Pyoderma Gangrenosum and Acne syndrome presented at 6 months of age and diagnosed at 4.5 years. He had recurrent pyogenic arthritis and skin abscesses and had a de novo and novel D246N mutation of *PSTPIP1*. He responded well to courses of prednisone.

Chronic Recurrent Multifocal Osteomyelitis (CRMO) group inlcuded 5 Arabic patients (2 males and 3 females) with a median age of disease onset of 7 years presenting with recurrent arthralgia (5), arthritis (3), abnormal gait (4) and back pain (2). One had compression fractures of the spine with kyphosis within 6 months of presentation. Other features included anemia (5) and psoriasis (1). All had elevated acute phase reactants, a diagnostic bone biopsy (3), bone scans (5), and MRI studies (5). Genetic testing results are pending in 2 whereas 2 had no *LPIN2* mutations but one had Q219H/- *PSTPIP1* variant. Treatments include naproxen (5), infliximab (3), pamidronate (2), and canakinumab (1).

## Conclusion

We report an expanding cohort of children with AID in Qatar. Clinical manifestations were variable for similar mutations even among the same family. Concomitant mutations in different AID genes can be present. Clinical phenotype of CRMO in our cohort was more severe then typically reported in the literature.

## Disclosure of interest

B. Aladbe: None declared, A. Aly: None declared, R. Taha: None declared, H. Elshanti: None declared, T. Moussa: None declared, F. AlAmry: None declared, B. Fathalla Grant / Research Support from: Medical Reserach Committee/ Hamad General Hospital

